# Nutrient Patterns and Their Food Sources in Older Persons from France and Quebec: Dietary and Lifestyle Characteristics

**DOI:** 10.3390/nu8040225

**Published:** 2016-04-19

**Authors:** Benjamin Allès, Cécilia Samieri, Simon Lorrain, Marthe-Aline Jutand, Pierre-Hugues Carmichael, Bryna Shatenstein, Pierrette Gaudreau, Hélène Payette, Danielle Laurin, Pascale Barberger-Gateau

**Affiliations:** 1 Centre INSERM U897-Epidemiologie-Biostatistique, University of Bordeaux, ISPED, Bordeaux, F-33000, France; cecilia.samieri@isped.u-bordeaux2.fr (C.S.); Simon.Lorrain@isped.u-bordeaux2.fr (S.L.); marthe-aline.jutand@isped.u-bordeaux2.fr (M.-A.J.); Pascale.Barberger-Gateau@isped.u-bordeaux2.fr (P.B.-G.); 2 Centre INSERM U897-Epidemiologie-Biostatistique, INSERM, ISPED, Bordeaux, F-33000, France; 3 Québec Center of Excellence on Aging, CHU de Québec Research Center, Quebec City, QC G1S 4L8, Canada; pierre-hugues.carmichael.cha@ssss.gouv.qc.ca (P.-H.C.); danielle.laurin@pha.ulaval.ca (D.L.); 4 Faculty of Pharmacy, Laval University, Quebec City, QC G1V 0A6, Canada; 5 Département de Nutrition, Université de Montréal, Montréal, QC H3T 1A8, Canada; bryna.shatenstein@umontreal.ca; 6 Centre de Recherche, Institut Universitaire de Gériatrie de Montréal, CIUSSS du Centre-est-de-l’Île-de-Montréal, Montréal, QC H3W 1W5, Canada; 7 Department of Medicine, University of Montreal, Montreal, QC H3C 3J7, Canada; pierrette.gaudreau@umontreal.ca; 8 Centre Hospitalier de l’Université de Montréal Research Center (CRCHUM), Montréal, QC H2X 0A9, Canada; 9 Research Center on Aging—Centre Intégré Universitaire de Santé et des Services Sociaux de l’Estrie—Centre Hospitalier Universitaire de Sherbrooke (CIUSS de l’Estrie-CHUS), Sherbrooke, QC J1H 4C4, Canada; Helene.Payette@usherbrooke.ca; 10 Faculty of Medicine and Health Sciences, University of Sherbrooke, Sherbrooke, QC J1K 2R1, Canada

**Keywords:** diet, nutritional quality, aged, nutrition, socioeconomic factors

## Abstract

*Background*: Dietary and nutrient patterns have been linked to health outcomes related to aging. Food intake is influenced by environmental and genetic factors. The aim of the present study was to compare nutrient patterns across two elderly populations sharing a common ancestral cultural background, but living in different environments. *Methods*: The diet quality, lifestyle and socioeconomic characteristics of participants from the Three-City Study (3C, France, *n =* 1712) and the Québec Longitudinal Study on Nutrition and Successful Aging (NuAge, Quebec, Canada, *n =* 1596) were analyzed. Nutrient patterns and their food sources were identified in the two samples using principal component analysis. Diet quality was compared across sample-specific patterns by describing weekly food intake and associations with the Canadian Healthy Eating Index (C-HEI). *Results*: Three nutrient patterns were retained in each study: a healthy, a Western and a more traditional pattern. These patterns accounted for 50.1% and 53.5% of the total variance in 3C and NuAge, respectively. Higher education and non-physical occupations over lifetime were associated with healthy patterns in both studies. Other characteristics such as living alone, having a body mass index lower than 25 and being an ex-smoker were associated with the healthy pattern in NuAge. No association between these characteristics and the nutrient patterns was noted in 3C. The healthy and Western patterns from each sample also showed an inverse association with C-HEI. *Conclusion*: The two healthy patterns showed important similarities: adequate food variety, consumption of healthy foods and associations with common sociodemographic factors. This work highlights that nutrient patterns derived using *a posteriori* methods may be useful to compare the nutritional quality of the diet of distinct populations.

## 1. Introduction

Nutrition is known to play a role in healthy aging. Numerous epidemiological and clinical studies have reported the benefits of specific nutrients, taken individually, in reducing the risk of chronic diseases in older persons [1,2]. However, this approach does not take into account the concept of food synergy, implying that a nutrient is never consumed alone and is interacting with many other nutrients or molecules [3]. The identification of dietary and nutrient patterns, which better reflect the complexity of dietary intake, to investigate the relationship between health and nutrition could overcome this limitation. Dietary patterns or the combinations of foods and beverages in diets, and nutrient patterns or the combinations of nutrients derived from data collected through dietary surveys, take into account the antagonist, additive and synergistic effects within the “food matrix” [3,4]. Only a few studies investigated nutrients taken as a whole, using dietary and nutrient patterns [5]. As reported in a study from the European Prospective Investigation into Cancer and Nutrition (EPIC), nutrients may characterize specific nutritional profiles enhancing comparisons between populations [6]. The EPIC has derived from food frequency questionnaire (FFQ) nutrient patterns within general European populations, highlighting that it was a better way to compare dietary intake from international populations than dietary patterns [6].

In recent reviews [7,8,9], healthy dietary or nutrient patterns characterized by higher consumptions of fruits and vegetables have been related to lower risks of cancer, diabetes, cardiovascular disease and Alzheimer’s disease, whereas Western or unhealthy patterns have been associated with increased risks. These reviews reported only a few studies investigating the relationships between the dietary or nutrient patterns and sociodemographic characteristics or the nutritional quality of the diet. Indeed, dietary and nutrient patterns are influenced by sociodemographic factors such as age, sex, socioeconomic status, and lifestyle [10]. In fact, among those factors, the Academy of Nutrition and Dietetics notes that food habits are especially determined by factors as living arrangements, finances, transportation, and disability [11]. Concerning the role of socioeconomic position in diet quality, the main hypothesis is that a higher socioeconomic status allows access to a more balanced diet than a lower status, but discordant results have been reported in some studies [12,13,14,15], and in other European countries and the US [10,16,17]. In Canada, individuals in the highest socioeconomic classes have the highest consumption of fruits and vegetables, following an income-education socioeconomic gradient [13]. Similar results have been observed in France [15]. Eating habits, diet quality and even nutritional risk in older people are influenced by living arrangement, especially loneliness or being widowed, but inconsistently [2,12,18].

To our knowledge, no comparison of dietary or nutrient patterns derived from two distinct populations of older people has been published yet. Additionally, no comparison studies have been conducted in two populations sharing a common ancestral cultural background. The aim of this study was to derive and validate nutrient patterns in two samples of older persons living in France and in Quebec, Canada. The nutritional quality of the diets of both study samples was described using nutrient intakes and their food sources. Finally, the link between nutrition and sociodemographic factors was explored and some comparisons between France and Quebec were enabled by this data harmonization.

## 2. Experimental Section

### 2.1. Study Populations

*The 3C study:* The French 3C study is a 12-year longitudinal cohort study of vascular risk factors for dementia which included 9294 community dwellers in Bordeaux (*n =* 2104), Dijon (*n =* 4931) and Montpellier (*n =* 2259), France. Individuals aged 65 years and over living in the community were randomly selected from electoral rolls. Methodological details of the study have been described elsewhere [19]. Baseline examination was carried out in 1999–2000; subjects were re-evaluated in 2001–2002 (wave 1) [19]. At baseline, standard data collection included sociodemographic and lifestyle characteristics, medical history, comprehensive neuropsychological testing, physical examination and blood sampling. In wave 1, there were 1712 participants (participation rate of 81.4%) from the Bordeaux sample asked to answer an extensive dietary survey conducted by trained dieticians, which included a 24-h dietary recall (excluding weekend days) and a food frequency questionnaire (FFQ). The present study is based on data from 3C wave 1 in Bordeaux.

*NuAge study:* The NuAge study is a four-year longitudinal study designed to evaluate the role of nutrition on physical and cognitive status, functional autonomy and social participation [20]. The study included 1793 individuals aged 67–84 years at baseline in 2003–2005. Participants in good general physical health and cognitively normal (Modified Mini-Mental State Examination score above 80 out of 100) were selected from a random sample obtained from the Quebec Medicare database using stratified sampling by age and sex [21]. After baseline examination, participants were re-examined annually over a three-year period. Trained dietitians and nurses assessed participants in face-to-face interviews or by direct measurement, at the research centers. Dietary data were collected through a FFQ at baseline and three annual 24-h dietary recalls. Further methodological details have been published elsewhere [20].

### 2.2. Dietary Assessments, Harmonization of Nutrient and Food Intake

In 3C, individual nutrient intakes were computed from the 24-h recall using the BILNUT^®^ software (Cerelles, France), which converts food intake data into nutrient intake data using French food composition tables [22]. These tables were augmented for fatty acids from the Food Composition and Nutrition tables [23]. As the 24-h recall was open-ended, additional data were also obtained by consulting a French table developed by the National Institute of Health and Medical Research (Inserm) and the University of Montreal [24], the USDA National Nutrient Database, food packaging, and directly contacting food manufacturers. Extended information about the dietary habits of the Bordeaux sample have been described in detail elsewhere [25,26]. Moreover, a qualitative FFQ, including 148 food and non-alcoholic beverage items, was administered during the same dietary survey [26]. Based on the FFQ, weekly frequency of consumption of 40 categories of foods and beverages for each of the three main meals and three between-meal snacks was recorded in 11 classes. The FFQ data were used to compare the frequency of food group consumptions between different nutrient patterns, and could not be used to estimate nutrient intake because portion size was not assessed.

In NuAge, daily nutrient intake levels were computed from the first of the three 24-h recalls at baseline using the CANDAT-Nutrient Calculation System (version 10, London, ON, Canada) based on the 2005 version of the Canadian Nutrient File, augmented by a database of 1200 additional foods that was developed on-site [27]. A 78-item validated semi-quantitative FFQ [28] was also administrated at recruitment to assess usual food intake. As in 3C, FFQ data enabled description of average food intake within each nutrient pattern.

Eleven food groups based on common food items to the two studies were defined. Nutrient-dense beverages such as sugary drinks were included in the sweet products group. As water and other hot beverages are not nutrient-dense beverages, they were not considered as contributors to nutrient intake. In addition, 21 nutrients whose consumption was available in 3C and NuAge were selected to derive nutrient patterns in both studies.

### 2.3. Nutritional Quality of the Diet

The Canadian Healthy Eating Index (C-HEI), based on the Healthy Eating Index (HEI) [29], a nine-item index, was used to assess diet quality using adherence to dietary guidelines in both samples. The C-HEI is based on intake of four food groups: grain products, fruits and vegetables, milk products, meat and alternatives, and five other items: % of energy as total fat intake and saturated fat intake, cholesterol, salt and diet variety [30]. The score ranges from 0 to 100, with higher scores indicating whether the nutritional quality of the diet is closer to the Canadian guidelines for healthy eating [30].

### 2.4. Data Harmonization for Sociodemographic and Lifestyle Variables

In both studies, living arrangement was categorized according to two modalities: living alone *vs.* in a couple or cohabitation. Smoking status was classified as current, ex-smoker, or never smoker. Education was categorized as 0–6 years, 7–9 years, 10–13 years and ≥14 years.

In 3C, monthly income was reported in four pre-determined categories according to the French economic situation in 1999–2000 [25]. Yearly income was further computed (converting French francs, the past French currency, to Euros), and created a three-category variable: low income (<18,000 euros (€)/year), moderate income (18,000–27,000 €/year) and high income (≥27,000 €/year). In NuAge, income was reported as a continuous variable. Because of differences in cost of living in 1999–2000 in France (baseline 3C) *versus* in 2003–2005 in Quebec (baseline NuAge), income values were not directly comparable. To allow comparability, the validated Big Mac Index was used [31] to convert income categories from 3C to corresponding categories in NuAge (<17,040 Canadian dollar (CAD), 17,040–25,560 CAD and ≥25,560 CAD). This conversion tool uses values of a McDonald’s Big Mac hamburger [31,32] at a given time in a given country, a food item consumed in many countries worldwide. Participants’ main occupation during their active years was collected according to the French occupation classification (International Standard Classification of Occupations 1988 (ISCO88)) [33] in 3C and the national Canadian occupation classification in NuAge [34]. In both studies, a variable was created indicating whether the occupation was physical (manufacturing, mining, and construction workers, *etc.*), non-physical (administration, trade managers, *etc.*) or mixed (arts, culture, sales, *etc.*). Body mass index (BMI) was computed as the weight (kg)/height squared (m^2^).

In 3C, missing data in food intake from the FFQ (66 participants, 3.7% of the sample) were imputed using Multivariate Imputation as described by Samieri *et al.* [35]. With respect to NuAge, 92 implausible FFQs among 1688 subjects (5%) with both 24-h recall and FFQs identified using a set of criteria developed by Shatenstein *et al.* [36] were excluded, leaving 1596 subjects for the final analysis. In both studies, missing values for covariates representing less than 5% of the sample were excluded, and dummy variables were created for variables with a frequency of missing values between 5% and 10%.

### 2.5. Statistical Analyses

Nutrient patterns are obtained using principal component factor analysis (FA-PCA). This procedure is performed on the correlation matrix of the 21 standardized nutrients common to both studies. In each sample, three principal components representing three independent nutrient patterns were identified according to their eigenvalues using scree plot to assess Cattel’s criterion (all eigenvalues for chosen factors above 1), interpretability and percentage of variance explained. Varimax rotation was performed to improve the interpretability of the factor loadings [37]. Component scores are obtained from the computed nutrient patterns and adjusted for energy using the residual method of Willett and Stampfer [38]. As a consequence, these component scores have an average of 0 and subjects who consumed less than the average on the defining nutrients would have a negative component score. Tucker’s congruence coefficient was computed to compare statistical similarities between the two sets of nutrient patterns [39]. A coefficient close to 1 signals a very high degree of similarity between two nutrient patterns; an accepted cut-off for good congruence is greater than 0.85.

Mean nutrient intake and food groups across quartiles of the obtained nutrient patterns were described for the two study samples. The relationship between the C-HEI, the dependent variable, and the component scores was assessed using separate linear regression adjusted for sex, following bivariate analyses of variance (ANOVA). Multivariate linear regression models were adjusted to assess the relationships between nutrient pattern scores and lifestyle characteristics in each sample controlling for age. Furthermore, the relationships between socioeconomic characteristics and nutrient pattern scores were compared between the two samples using interaction terms in a single model.

## 3. Results

Compared with 3C participants, those from NuAge were significantly younger, more likely to be men and showed a lower proportion of subjects with six or less years of education (Table 1). NuAge participants were significantly less likely to be in the lower income category and more likely to report a non-physical occupation over lifetime and to be living as a couple or in cohabitation, suggesting they had a better socioeconomic position than those in 3C. Significantly higher BMI values and energy intake for both men and women were found in the NuAge study, as well as a higher proportion of ex-smokers.

In 3C, the FA-PCA yielded a three-component solution that accounted for 50.1% of the total variance (Table 2). The first component explained 21.6% of the variance, and was characterized by, in decreasing order, higher intake of potassium, dietary fiber, magnesium, folates, vitamin B_6_, carbohydrates, vitamin C, iron, vitamin E and carotene (all factor loadings >0.20). The second component explained 18.6% of the variance, and reflected high intake of monounsaturated fatty acids (MUFA), saturated fatty acids (SFA), phosphorus, proteins, *n*-3 polyunsaturated fatty acids (PUFA), calcium, *n*-6 PUFA, vitamin D and vitamin E (equally loaded between components 1 and 2). The third component with 9.9% of the explained variance was related to vitamin B_12_ and vitamin A intake. The mean nutrient intakes across quartiles of each factor score are described in Table S1. As expected, increasing intakes across quartiles of factor score were observed for nutrients with positive factor loadings from FA-PCA. Additionally, the third nutrient pattern had the highest consumption of alcohol.

In NuAge, the FA-PCA yielded also a three-component solution that accounted for 53.5% of the total variance (Table 2). The first component explained 22.8% of the variance, and was related, in decreasing order, by high intake of potassium, magnesium and dietary fiber (equally loaded), vitamin B_6_, vitamin C, phosphorus, iron and carbohydrates (equally loaded), vitamin E and carotene (equally loaded). The second component accounted for 19.2% of the variance and was related to high intake of MUFA, SFA, *n*-3 PUFA, proteins, folates, calcium, *n*-6 PUFA and vitamin D. The third component with 11.5% of the explained variance was related to high intake of vitamin B_12_, vitamin A and zinc. Similarly to the 3C study, increasing intake across quartiles of factor score were observed for nutrients with positive factor loadings from FA-PCA (Table S2).

The congruence coefficient between the first nutrient patterns of both 3C and NuAge was 0.83, indicating a borderline similarity. A moderate similarity (congruence coefficient of 0.76) was found between the second nutrient patterns of both samples, and a low similarity (congruence coefficient of 0.60) was found between the third nutrient patterns.

Mean intakes of food groups were described according to quartiles of energy-adjusted factor scores for each factor in each sample. In 3C (Table 3), the first pattern was significantly associated with greater consumption of vegetables, legumes, fruits, cereals, potatoes, fish and seafood, and with lower intake of charcuterie and alcohol. This pattern showed a relatively balanced diet and was therefore labelled healthy. Conversely, higher score on the second pattern was associated with higher intake of charcuterie and dairy products, and lower intake of vegetables, legumes, fruits, cereals, potatoes, biscuits and other sweet foods and alcohol. This pattern was labelled Western. The third pattern characterized by high intake of meat, charcuterie, fish/seafood and alcohol was typical of Bordeaux regional food behavior. Accordingly, this third pattern was labelled “traditional—South-West of France”. This pattern is also characterized by lower intake of dairy products and biscuits/sweet foods.

Similar results were observed in NuAge (Table 4). The first pattern, significantly associated with higher intake of vegetables, legumes, fruits, cereals, fish/seafood, and dairy products, and with lower intake of potatoes, charcuterie, and biscuits and other sweet foods, was labelled healthy. Conversely, the second pattern associated with lower consumption of vegetables, legumes, fruits, fish/seafood, and alcohol, and higher intake of biscuits and other sweet food, was labelled Western. A third pattern was associated with higher intake of fish/seafood and dairy product intakes, and lower intake of biscuits and sweet food consumption. Considering the customary consumption of fish/seafood products on Fridays by older people in Quebec, it was labelled traditional.

**Figure 1:**
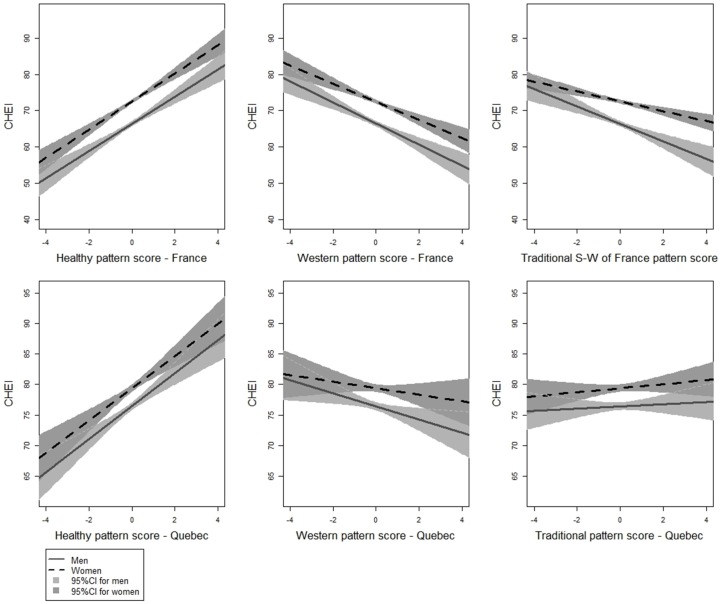
Linear relationships between energy-adjusted nutrient pattern scores and C-HEI scores by sex (*n*_3C_ = 1712. *n*_NuAge_ = 1596)*. * For reasons of clarity, 21 subjects in 3C and 12 subjects in NuAge are not presented in their respective traditional pattern score plots.

Sex-specific associations between factor scores and the C-HEI for both 3C and NuAge models are shown in Figure 1 (*r*^2^ = 0.35 in 3C and 0.15 in NuAge in models adjusted for sex (both *F* test *p*-values < 0.05)). In both studies, higher healthy pattern scores were associated with higher adherence to the C-HEI. No interaction was detected between sex and nutrient pattern scores on C-HEI scores. The mean increase in C-HEI score for each increase of 1 unit of the healthy pattern score was 3.88 in 3C (95% confidence interval (CI) = (1.68, 5.58)), and 2.70 in NuAge (95% CI = (1.85, 3.54)). Conversely, there was a significant inverse relationship between C-HEI scores and factor scores for Western patterns in both studies. The mean decrease in C-HEI score for each increase of 1 unit of the Western pattern score was −2.51 in 3C (95% CI = (−5.29, −1.34)), and −1.07 in NuAge (95% CI = (−1.92, −0.23)). The traditional South-West of France pattern in 3C was inversely associated with C-HEI: the mean decrease in C-HEI for each increase of 1 unit of the traditional pattern score was −1.36 (95% CI = (−5.38, −1.56)). The traditional pattern in NuAge was not associated with the C-HEI score. Compared to men, women showed higher C-HEI scores for every nutrient pattern (β_women-3C_ = 6.13, 95% CI = (5.21, 7.04) and β_women-NuAge_ = 2.98, 95% CI = (2.09, 3.87)).

Associations between socioeconomic, health and lifestyle characteristics and the nutrient patterns were examined in both studies. In 3C, results from bivariate analyses were confirmed in multivariate models for each of the nutrient pattern scores (Table 5). Indeed, the same statistically significant associations were observed except for main occupation in the Western pattern. The healthy pattern was significantly associated with higher education (only statistically significant for the 10–13 years of education category), whereas the Western pattern was significantly associated with female sex. No association was found for yearly income, living arrangement, BMI and smoking status. In NuAge (Table 6), results from bivariate analysis were confirmed in multivariate linear models for each nutrient pattern score, with the exception of occupation, which was not associated with the Western pattern in multivariate model. The healthy pattern was significantly positively associated with higher education (only statistically significant for the 14+ years of education category), lower BMI, and non-physical occupation, and negatively associated with living in a couple or cohabitation and smoking. The Western pattern was significantly associated with smoking, lower education and higher BMI.

When comparing the relationships between the two studies, significant interactions were found for education (*p* < 0.01 for Healthy pattern), main occupation during lifetime (*p* < 0.01 for Healthy pattern and *p* = 0.02 for Western pattern), sex (*p* *<* 0.01 for Western pattern) and BMI (*p* < 0.01 for Healthy pattern and *p* = 0.03 for Western pattern).

## 4. Discussion

This study identified distinct nutrient patterns in older persons living in France and in Quebec, Canada: a healthy pattern and a Western pattern, both relatively similar in the two populations in terms of nutrient/food characteristics, especially for the healthy pattern. In both studies, the healthy pattern was reflecting good food variety and balance. Conversely, in both studies, there was a poorer variety of nutrients in the Western pattern. Very few nutrients were highly loaded in each traditional pattern, but further examination of food consumption and nutritional quality of the diet led to description of specific underlying food habits. In this study, nutrient patterns rather than dietary patterns allowed us to characterize and compare more efficiently nutritional quality of the diet of both study samples as nutrient intakes were assessed in each study using French and Canadian food composition tables, respectively.

In the 3C study sample, we labelled the second dietary pattern “Western” although this pattern may be less characteristic of a Western diet, compared to the Western dietary pattern described by Popkin [40] and identified, for example, by Hu *et al.* in an epidemiological study [4]. However, similar to the findings of Hu *et al.*, nutrients in fruits and vegetables such as fibers, carotene and vitamin C have low loadings on this Western pattern, which is in opposition to a prudent or healthy pattern. *A posteriori* dietary patterns are dependent on the sample of analysis, limiting external validity [9]. Although the Western pattern identified in 3C has a different quality of the diet compared to Western patterns from previous studies, it could still be characteristic of a Western diet within our two samples.

Surprisingly, nutrient patterns found in the current study sample from NuAge using data from a 24-h recall (*n =* 1596) differ in some ways from dietary patterns identified in another sample from NuAge using data from the FFQ [41]. In their analysis, Parrott *et al.* [41] reported dietary patterns that were labelled “prudent” and “Western”. The two dietary patterns accounted for 10.4% of the total variance of the diet, contrasting with our three nutrient patterns accounting for 53.5% of the variance. These discrepancies may be related to several methodological issues. In our study, we used quantitative nutrient intake variables from 24-h recall to derive nutrient patterns, whereas Parrott *et al.* [41] used qualitative food intake variables from FFQ in a smaller sample to derive dietary patterns. In contrast to the latter study, we adjusted the nutrient patterns for energy intake, a major confounding factor for dietary patterns.

Previous work by Samieri *et al.* within the 3C cohort allowed the identification of five clusters of dietary patterns [35]. In their analysis, a healthy dietary pattern was reported associated with higher education and higher income. Even if comparison is limited because the dietary patterns were derived from FFQ in their study, this result suggests a good internal validity of our results.

A study from the EPIC cohort also studied the relationship between nutrient patterns and their food sources, and their association with sociodemographic characteristics [6]. Among the four nutrient patterns obtained in this study, the nutritional quality of the diet of the second pattern seemed similar to the healthy pattern derived in our study. Indeed, the healthy dietary pattern from the EPIC study was associated with higher consumption of fruits, vegetables and fish. This nutrient pattern was associated with a higher level of education. The similarities between these results from the EPIC study and ours, in adequation with hypotheses from previous studies [13,15], highlight that level of education might play a role in nutritional quality of the diet.

Two dietary patterns with inverse quality have been previously reported in other studies of elderly people. This duality was described among a sample of 205 American older persons with a mean age of 78 years from the Geisinger Rural Aging Study (GRAS) [42], using 24-h records as dietary assessment. The authors reported a prudent pattern characterized by high intake of fruits, vegetables, white meat, dairy and whole grain products and low consumption of fried fish. In that study, the Western pattern consisted of high consumption of sweets and candy, processed meats and salty snacks. The prudent pattern was strongly positively correlated with a higher mean adequacy ratio of essential nutrients, whereas the Western pattern was inversely correlated with this ratio. Correlations with global nutritional quality of the diet indices were not reported.

In a sample of 4693 older people (mean age 60 years) from the UK Whitehall II study, using dietary data from a FFQ, Akbaraly *et al.* [43] also identified a whole food pattern and a processed food pattern, similar to the healthy and Western patterns identified in our study. As in the NuAge sample, higher adherence to the whole food pattern was associated with a higher level of education and being ex-smokers. The reverse was true for the processed food pattern.

Similar findings were also reported by Kesse-Guyot *et al.* [44] in a French population of 3054 subjects studied at midlife, aged on average 52 years old at nutritional assessment. The authors described a healthy pattern derived from dietary data from a 24-h dietary record that was also correlated with high consumption of fruits, dairy products, vegetables, and fish. This healthy pattern was inversely correlated with meat and processed meat, which is similar to the healthy patterns from both 3C and NuAge samples.

Food habits in South-West of France, leading to the phenomenon known as the French paradox [45], are typically related to consumption of charcuterie and wine as well as fish/seafood, especially in the Bordeaux area due to the proximity of the Atlantic Ocean. Thus, a traditional food pattern has been previously reported composed of both recommended and non-recommended food and associated nutrients [46].

In Quebec, the traditional food pattern was related to higher intake of dairy products and fish, which used to be part of their traditional diet. This pattern may be explained in part by an advertising campaign promoting dairy products for the prevention of osteoporosis, a wider distribution of fish to markets and publicity for the prevention of cardiovascular disease and memory deficits with advancing age. However, this dietary pattern explicated a small amount of the total variance.

To assess nutritional quality of the diet, we chose the C-HEI because items necessary to compute this score are based on food groups and nutrient intake data that were available in both NuAge and 3C samples. Thus, the healthy patterns appeared to be associated with healthy food habits in each country, in line with the Canadian dietary guidelines, whereas the Western patterns may reflect an unbalanced diet with low adherence to these guidelines. Among those following the traditional pattern in 3C, adherence to these healthy eating guidelines was moderate, whereas in Quebec, the traditional pattern was associated with a better adherence. In NuAge, participants reported a higher weekly consumption of vegetables and lower consumption of charcuterie. This resulted in lower saturated fat intakes and may explain the higher adherence to dietary guidelines observed among those characterized by the traditional pattern.

Greater adherence to Canadian dietary guidelines was found in women but no statistical interactions on C-HEI score were observed, thus sex was not a modifier of this association and may be a potential confounder. Although it has been suggested that sex differences are important in dietary pattern analyses [41,47], our results do not support this evidence.

Socioeconomic position, lifestyle and other habits such as smoking are potentially associated with nutrient patterns. In both 3C and NuAge, higher education was associated with a healthy pattern as found in some studies [13,15,41]. However, comparisons between the two samples revealed that the association between higher education and healthy pattern seemed to be stronger in the NuAge sample (data not shown). We also compared the results of the associations between healthy pattern and additional socio-economic characteristics in the two samples. They were associated with healthy pattern only in NuAge, suggesting that socioeconomic position may have a significant influence on dietary choices in this population, similar to the results from Parrott *et al.* [41]. People with a higher level of education may have better nutrition knowledge and greater earning power, even though income was not statistically associated in our study, allowing them to choose expensive healthy food [16,48]. These findings are altogether consistent with the hypothesis that nutritional quality of the diet is associated with socioeconomic position. Higher education may play a greater role among these factors as it has been found associated with nutritional quality of the diet in both samples from our study.

In NuAge, the Western pattern was associated with higher BMI values but factor scores were adjusted for energy intake suggesting that that diet probably yielded this association. Mean total energy intake was 20% to 25% higher in NuAge than in 3C sample. Mean energy intake in NuAge reach Canadian recommendations for sedentary people under 71 but are above recommendations for people 71 years and over [49]; this may explain higher BMIs in this sample but could also be due to higher proportion of men in the NuAge sample. However, the cross-sectional design of this study does not allow us to ascertain temporality of the relationship between BMI and identified patterns.

Some limitations to our findings must be recognized. One of them is that dietary data were based on a single 24-h recall. However, it has been suggested that a single recall can provide an accurate estimation of average nutrient intake in large study samples [50]. In order to improve comparability between studies, we decided to use only the first 24-h recall to derive nutrient patterns in NuAge as in 3C. Analyses showed that percentage of explained variance of diet from the three nutrient patterns in NuAge was very similar when using three 24-h recalls (data not shown). Regarding the FFQ, for a same food group, we gathered frequencies of consumption of food items that were not estimated using the same method. For example, for the vegetable food group in NuAge, there were 13 items asking for separate categories of the most frequently consumed vegetables (beans, tomatoes, carrots, salad, *etc.*), whereas in 3C there were 12 items of vegetable consumption separated into two items for raw and two items for cooked vegetables, for each of the three meals. This may have introduced an information bias that could have underestimated vegetable consumption in the 3C sample. Conversely, closed lists of food items in FFQs as in NuAge tend to overestimate consumption. Thus, numbers of servings per week are not directly comparable between the two samples. This was an additional reason for deriving nutrient-based rather than food-based patterns. The qualitative FFQ in 3C did not allow us to describe quantities in food intake.

Although the FA-PCA allowed the identification of three dietary patterns, the aim of this method is not to classify subjects into distinct groups of nutrient intake. Indeed, individuals that have very similar intakes of different major food groups and are classified as belonging to one of the latent nutrient categories would also belong at the low end of a different latent category. Thus, the result of this analysis should be interpreted with caution.

To our knowledge, this is the first analysis using nutrient intake data from two observational studies. As mentioned, France and Quebec have a common ancestral cultural background, but Quebec has further developed food habits influenced by North American culture. Indeed, according to historians, as people from Quebec were French settlers, they inherited French food habits during the 17th century and were lately influenced by English Canadian people during late 18th century and beginning of the 19th century [51]. Thus, their diet is now a mix of those cultures. This may partially explain why Quebec and South-West of France appear to share common healthy food habits as opposed to a Western pattern characterized by lower food diversity. This research addressed identification of potential confounders of nutrient patterns as well as data harmonization between these two cohorts in order to further contribute to the investigation of associations between nutrient patterns and health outcomes. Moreover, this study highlights the need to choose the best source of dietary data to derive nutritional patterns. In fact, dietary assessment method (e.g., FFQ *vs.* 24-h recall) may also change the resulting dietary patterns within a population. Thus, describing characteristics of nutrient patterns instead of food patterns to compare nutritional quality of the diet between populations may be the best method to assess reproducibility of the results, an important part of causality in studies.

## 5. Conclusions

Overall, our findings add to previous literature suggesting, in various Western populations, a relatively consistent opposition between healthy and Western patterns. Further investigations are needed to enhance the methodology of dietary comparisons between populations. Finally, comparisons of the nutritional quality of the diet between countries may enhance the internationalization of dietary guidelines among populations sharing a common ancestral cultural background.

## Figures and Tables

**Table 1:** Characteristics of the 3C study sample (*n =* 1712) and the NuAge study sample (*n =* 1596).

	3C		NuAge		*p* *
**Age, years (mean SD)**	76.5	5.1	74.3	4.2	<0.001
**Sex (*n* %)**					
Men	644	37.6	763	49.1	<0.001
Women	1068	63.4	833	51.9	
**Education, years (*n* %) ^†^**					
0–6	227	13.3	156	9.8	<0.001
7–9	376	22.0	402	25.1	
10–13	458	26.8	547	34.3	
14+	650	38.0	491	30.8	
**Yearly income (*n* %)**					
Missing or refused to answer	138	8.1	155	9.7	<0.001
Lower income (<17,040 CAN$)	645	37.7	315	19.7	
Moderate income (17,040 to 25,560 CAN$)	426	24.9	914	57.3	
Higher income (>25,560 CAN$)	503	29.4	212	13.3	
**Main occupation over lifetime (*n* %) ^‡^**					
Physical occupation	466	27.3	392	24.7	<0.001
Non-physical occupation	495	28.9	699	44.0	
Mixed occupation	749	43.8	497	31.3	
**Living arrangement (*n* %) ^§^**					
Alone	917	53.7	505	31.7	<0.001
Couple or cohabitation	791	46.3	1090	69.3	
**BMI (*n* %)**					
BMI < 25	744	43.5	431	27.0	<0.001
25 ≤ BMI < 30	700	40.9	759	47.6	
BMI ≥ 30	268	15.7	406	25.4	
**Energy intake (mean SD)**					
Men	1706	539	2131	672	<0.001
Women	1517	457	1726	540	
**Smoking (*n* %) ^ǁ^**					
Non-smoker	1051	61.6	835	52.3	<0.001
Ex-smoker	552	32.3	654	41.0	
Smoker	104	6.1	107	6.7	

BMI: Body mass index, SD: standard deviation; * *p* for χ^2^ for categorical variables and *t* test for linear variables; BMI: Body mass index, SD: standard deviation; ^†^ 1 missing value for 3C; ^‡^ 2 missing values for 3C, 8 for NuAge; ^§^ 4 missing values for 3C, 1 for NuAge; ^ǁ^ 5 missing values for 3C.

**Table 2:** Nutrient patterns obtained by factor analysis using principal component analysis of nutrient intakes.

3C				NuAge			
Nutrient	Factor 1 Healthy	Factor 2 Western	Factor 3 Traditional South-West of France	Nutrient	Factor 1 Healthy	Factor 2 Western	Factor 3 Traditional
Potassium	**0.83**	0.30	−0.01	Potassium	**0.86**	0.28	0.22
Fiber	**0.78**	0.10	−0.03	Fiber	**0.81**	0.17	0.02
Magnesium	**0.75**	0.46	−0.03	Magnesium	**0.81**	0.35	0.16
Folates	**0.70**	0.07	0.44	Vitamin B_6_	**0.73**	0.25	0.29
Vitamin B_6_	**0.67**	0.37	0.19	Vitamin C	**0.66**	−0.09	−0.04
Carbohydrates	**0.61**	0.34	−0.09	Carbohydrates	**0.57**	0.49	0.02
Vitamin C	**0.58**	−0.17	0.09	Phosphorus	**0.57**	0.52	0.39
Iron	**0.49**	0.34	0.35	Iron	**0.57**	0.48	0.28
Carotene	**0.42**	−0.17	0.00	Carotene	**0.48**	−0.10	−0.01
Zinc	**0.14**	0.08	0.08	Vitamin E	**0.48**	0.32	0.01
Vitamin E	**0.31**	**0.31**	0.07	MUFA	0.13	**0.86**	0.09
Proteins	0.47	**0.67**	0.05	SFA	0.00	**0.82**	0.16
MUFA	0.10	**0.77**	0.06	PUFA-*n*3	0.11	**0.68**	−0.09
SFA	0.10	**0.76**	0.03	Proteins	0.41	**0.57**	0.39
Phosphorus	0.51	**0.72**	0.04	Calcium	0.41	**0.46**	0.25
PUFA-n3	−0.06	**0.58**	0.06	Folates	0.00	**0.45**	−0.01
Calcium	0.32	**0.55**	−0.09	PUFA-*n*6	0.21	**0.40**	−0.11
PUFA-n6	0.19	**0.49**	0.09	Vitamin D	0.24	**0.27**	0.26
Vitamin D	−0.17	**0.42**	0.11	Vitamin B_12_	0.01	−0.04	**0.92**
Vitamin A	0.05	0.01	**0.91**	Vitamin A	0.02	−0.10	**0.83**
Vitamin B_12_	0.08	0.14	**0.91**	Zinc	0.25	0.42	**0.49**
Variance explained (%)	21.6	18.6	9.9		22.8	19.2	11.5

SFA: saturated fatty acids, MUFA: monounsaturated fatty acids, PUFA: polyunsaturated fatty acids.

**Table 3:** Mean food group intakes according to quartiles of component scores obtained by factor analysis—principal component analysis of nutrient intake data in 3C (*n =* 1712), from food frequency questionnaires.

	Healthy Pattern Score					Western Pattern Score					Traditional—South-West of France Pattern Score				
Food Groups (Servings Per Week)	Q1 (Lowest) *n =* 428	Q2 *n =* 428	Q3 *n =* 428	Q4 (Highest) *n =* 428	*p* *	Q1 *n =* 428	Q2 *n =* 428	Q3 *n =* 428	Q4 *n =* 428	*p* *	Q1 *n =* 428	Q2 *n =* 428	Q3 *n =* 428	Q4 *n =* 428	*p* *
Vegetables	16.5	18.5	20.1	21.7	<0.001	20.3	19.3	19.0	18.2	<0.001	18.7	19.4	19.5	19.1	0.38
Legumes	0.6	0.6	0.6	0.7	0.04	0.7	0.6	0.6	0.6	0.009	0.6	0.6	0.6	0.6	0.93
Fruits	11.1	12.6	14.3	16.1	<0.001	15.5	13.8	13.1	11.7	<0.001	13.9	14	12.5	13.6	0.006
Cereals	18.8	19.6	20.5	20.4	<0.001	20.7	20.5	19.5	18.6	<0.001	20.3	20	19.4	19.7	0.14
Potatoes	2.6	2.5	2.7	2.8	0.03	2.8	2.6	2.5	2.5	0.04	2.8	2.7	2.5	2.5	0.01
Meat	4.8	4.8	4.8	4.8	0.98	4.7	4.9	4.9	4.7	0.66	4.6	4.5	5	5	0.002
Fish/seafood	2.7	2.8	2.8	3.1	0.004	2.9	2.8	2.8	2.9	0.55	2.6	2.8	3.1	3	<0.001
Charcuterie	2.1	1.7	1.4	1.4	<0.001	1.5	1.7	1.5	1.9	0.04	1.5	1.5	1.7	2	<0.001
Dairy products	18.1	18.1	18	18.7	0.48	17.2	17.8	18.5	19.5	<0.001	20.3	18.5	17.5	16.5	<0.001
Biscuits and other sweet food	11.5	11.5	11.1	10.2	0.06	12.2	11.2	11.1	9.7	<0.001	11.7	11.6	10.9	10	0.001
Alcohol	14.4	11.7	11.2	12.6	0.02	15.8	13.4	9.9	10.8	<0.001	11	10.9	13.1	14.9	<0.001

* *p* for ANOVA test.

**Table 4:** Mean food group intakes according to quartiles of component scores obtained by factor analysis—principal component analysis of nutrient intake data in NuAge (*n =* 1596), from food frequency questionnaires.

	Healthy Pattern Score					Western Pattern Score					Traditional Pattern Score				
Food Groups (Serving per Week)	Q1 (Lowest) *n =* 399	Q2 *n =* 399	Q3 *n =* 399	Q4 (Highest) *n =* 399	*p* *	Q1 *n =* 399	Q2 *n =* 399	Q3 *n =* 399	Q4 *n =* 399	*p* *	Q1 *n =* 399	Q2 *n =* 399	Q3 *n =* 399	Q4 *n =* 399	*p* *
Vegetables	25.2	29.5	31.7	36.2	<0.001	35.2	31.5	28.8	27.1	<0.001	31	30.6	30.2	30.9	0.86
Legumes	0.7	1	1.1	1.4	<0.001	1.2	1	1.1	0.9	0.02	1.2	1	1	1	0.11
Fruits	11.2	13.1	15.8	18	<0.001	17.4	14.6	13.9	12.2	<0.001	15.2	14.3	14.3	14.3	0.18
Cereals	13.8	14.8	14.2	15.7	<0.001	15.2	14.4	14.5	14.4	0.11	14.2	15	14.7	14.7	0.35
Potatoes	4.2	3.8	3.9	3.3	<0.001	3.6	3.7	3.8	3.9	0.33	3.9	3.6	3.7	3.9	0.42
Meat	3	3.1	3	2.8	0.36	2.8	3	2.9	3	0.4	2.8	3	2.9	3.1	0.38
Fish/seafood	1.5	1.8	1.8	2.3	<0.001	2.2	1.8	1.7	1.6	<0.001	1.7	1.8	1.9	2	0.04
Charcuterie	1.8	1.5	1.5	1.2	<0.001	1.4	1.4	1.4	1.7	0.13	1.4	1.5	1.5	1.6	0.15
Dairy products	21.4	21.5	20.9	23.6	<0.001	21.7	22.4	21	22.4	0.44	19.4	20.8	22.5	24.8	<0.001
Biscuits and other sweet food	20.7	17.2	14.9	13.8	<0.001	16.2	16.1	16.1	18.1	0.03	17.3	17.6	16.5	15.1	0.02
Alcohol	3.8	3.8	3.5	3.9	0.97	4.9	4	2.8	3.2	<0.001	4	3.4	3.6	3.9	0.17

**Table 5:** Associations between social, health and lifestyle characteristics of the subjects and nutrient patterns in 3C (*n =* 1712).

	Healthy Pattern				Western Pattern				Traditional—South-West of France Pattern			
	Unadjusted		Adjusted		Unadjusted		Adjusted		Unadjusted		Adjusted	
	Mean Residual Score	*p* *	β	95% CI	Mean Residual Score	*p* *	β	95% CI	Mean Residual Score	*p* *	β	95% CI
**Sex**												
Men	0.018	0.58	Reference		−0.093	<0.001	Reference		−0.014	0.39	Reference	
Women	0.013		−0.03	−0.14; 0.08	0.046		0.12	0.03; 0.20	0.028		0.06	−0.06; 0.16
**Education**												
0–6 years	−0.134	0.01	Reference		0.059	0.24	Reference		−0.055	0.86	Reference	
7–9 years	−0.052		0.05	−0.09; 0.15	0.027		−0.02	−0.13; 0.09	−0.007		0.01	−0.16; 0.17
10–13 years	0.122		0.20	0.05; 0.35	−0.011		−0.02	−0.13; 0.09	0.016		0.04	−0.13; 0.20
14+ years	0.021		0.12	−0.03; 0.27	−0.046		−0.04	−0.16; 0.07	0.037		0.03	−0.15; 0.20
**Yearly income ^†^**												
Lower income	−0.154	0.23	Reference		0.094	0.15	Reference		0.111	0.36	Reference	
Moderate income	0.006		0.03	−0.09; 0.15	0.025		−0.02	−0.11; 0.07	−0.062		0.05	−0.08; 0.19
Higher income	0.058		−0.04	−0.18; 0.19	−0.025		−0.01	−0.11; 0.09	0.000		0.13	−0.03; 0.28
Missing or refused to answer	0.010		−0.11	−0.28; 0.05	−0.041		0.04	−0.09; 0.16	0.060		−0.04	−0.23; 0.15
**Main occupation over lifetime**												
Physical occupation	−0.045	0.15	Reference		0.023	0.03	Reference		0.004	0.86	Reference	
Non-physical occupation	−0.025		0.01	−0.09; 0.12	0.050		0.01	−0.07; 0.09	−0.020		−0.03	−0.16; 0.09
Mixed occupation	0.047		0.08	−0.03; 0.19	−0.046		−0.02	−0.11; 0.06	0.011		−0.02	−0.15; 0.11
**Living arrangement**												
Alone	0.017	0.43	Reference		−0.026	0.08	Reference		−0.005	0.77	Reference	
Couple or cohabitation	−0.017		−0.02	−0.12; 0.07	0.030		−0.01	−0.07; 0.07	0.008		0.05	−0.06; 0.16
**BMI**												
BMI ≥ 30	0.039	0.76	Reference		−0.018	0.94	Reference		−0.010	0.77	Reference	
25 ≤ BMI < 30	−0.010		−0.04	−0.14; 0.04	−0.007		0.02	−0.04; 0.09	0.028		0.05	−0.05; 0.15
BMI < 25	0.028		0.01	−0.10; 0.14	0.009		0.01	−0.07; 0.11	0.013		0.04	−0.10; 0.19
**Smoking**												
Non-smoker	0.036	0.76	Reference		0.016	0.14	Reference		0.030	0.89	Reference	
Ex-smoker	−0.007		0.07	−0.11; 0.25	−0.055		0.01	−0.12; 0.14	−0.021		−0.01	−0.21; 0.20
Smoker	−0.072		0.01	−0.18; 0.18	0.001		0.02	−0.11; 0.16	0.004		−0.01	−0.22; 0.20

* *p* for *t* test for sex and ANOVA for other variables; ^†^ Lower income: <18,294 €, Moderate income: 18,294 to 27,440 €, Higher income: >27,440 €; Bivariate analyses were performed using mean residual score separately for each social, health or lifestyle variables.; Multivariate linear models were adjusted for social, health and lifestyle (all variables presented in this table) variables altogether in a same model.

**Table 6:** Associations between social, health and lifestyle characteristics of the subjects and nutrient patterns in NuAge (*n =* 1596).

	Healthy pattern				Western pattern				Traditional pattern			
	Unadjusted		Adjusted		Unadjusted		Adjusted		Unadjusted		Adjusted	
	Mean Residual Score	*p* *	β	95% CI	Mean Residual Score	*p* *	β	95% CI	Mean Residual Score	*p* *	β	95% CI
**Sex **												
Women	0.033	0.13	Reference		0.017	0.23	Reference		0.001	0.98	Reference	
Men	−0.036		0.03	[−0.08; 0.14]	−0.020		0.05	[−0.01; 0.13]	−0.001		0.06	[−0.06; 0.18]
**Education**												
0–6 years	−0.175	<0.001	Reference		0.034	<0.001	Reference		−0.014	0.22	Reference	
7–9 years	−0.169		−0.02	[−0.19; 0.14]	0.083		0.04	[−0.06; 0.15]	−0.082		−0.08	[−0.26; 0.11]
10–13 years	−0.028		0.05	[−0.12; 0.22]	0.019		−0.01	[−0.11; 0.10]	0.052		0.07	[−0.11; 0.26]
14+ years	0.226		0.27	[0.09; 0.46]	−0.100		−0.12	[−0.23; −0.01]	0.014		0.05	[−0.15; 0.25]
**Yearly income ^†^**												
Lower income	−0.092	0.05	Reference		0.003	0.91	Reference		−0.045	0.36	Reference	
Moderate income	−0.068		0.08	[−0.08; 0.26]	0.013		−0.02	[−0.12; 0.09]	0.014		0.05	[−0.14; 0.24]
Higher income	0.055		0.11	[−0.05; 0.27]	−0.010		0.03	[−0.07; 0.14]	0.026		0.06	[−0.12; 0.23]
Missing or refused to answer	−0.068		0.01	[−0.18; 0.19]	0.015		0.01	[−0.09; 0.15]	−0.100		−0.06	[−0.27; 0.15]
**Main occupation over lifetime**												
Physical occupation	−0.219	<0.001	Reference		0.076	0.006	Reference		0.003	0.91	Reference	
Non-physical occupation	0.107		0.16	[0.03; 0.29]	−0.040		0.07	[−0.01; 0.16]	−0.009		−0.07	[−0.22; 0.07]
Mixed occupation	0.018		0.13	[−0.01; 0.27]	−0.010		0.02	[−0.05; 0.08]	0.015		−0.02	[−0.18; 0.13]
**Living arrangement**												
Alone	0.081	0.02	Reference		−0.020	0.50	Reference		0.009	0.80	Reference	
Couple	−0.037		−0.11	[−0.21; −0.01]	0.006		0.01	[−0.05; 0.08]	−0.005		−0.01	[−0.12; 0.11]
**BMI**												
BMI ≥ 30	0.137	<0.001	Reference		−0.070	<0.001	Reference		−0.017	0.4	Reference	
25 ≤ BMI < 30	−0.002		0.23	[0.11; 0.36]	−0.010		−0.16	[−0.24; −0.08]	0.034		0.04	[−0.07; 0.17]
BMI < 25	−0.142		0.12	[0.01; 0.23]	0.102		−0.10	[−0.17; −0.03]	−0.044		−0.03	[−0.17; 0.10]
**Smoking**												
Non smoker	0.008	0.001	Reference		0.010	0.03	Reference		−0.041	0.21	Reference	
Ex-smoker	0.030		−0.04	[−0.15; 0.04]	−0.030		0.04	[−0.18; 0.11]	0.051		0.05	[−0.15; 0.25]
Smoker	−0.247		−0.30	[−0.49; −0.12]	0.115		0.17	[0.05; 0.28]	0.007		0.09	[−0.01; 0.01]

* *p* for *t* test for sex and ANOVA for other variables; **^†^** Lower income: <17,040 CAD (Canadian dollars), moderate income: 17,040–25,560 CAD, Higher income: >25 560 CAD; Bivariate analyses were performed using mean residual score separately for each social, health or lifestyle variables; Multivariate linear models were adjusted for social, health and lifestyle (all variables presented in this table), variables altogether in a same model.

## Supplementary Materials

The following are available online at http://www.mdpi.com/2072-6643/8/4/225/s1, Table S1: Mean daily nutrient intakes according to quartiles of component scores obtained by factor analysis—principal component analysis of nutrient intake data in 3C (*n* = 1712) from 24-h recall, Table S2: Mean daily nutrient intakes according to quartiles of component scores obtained by factor analysis—principal component analysis of nutrient intake data in NuAge (*n* = 1596) from first 24-h recall.
