# Supplementary material for: Nutrient Patterns and Their Food Sources in Older Persons from France and Quebec: Dietary and Lifestyle Characteristics
Source: Nutrients. 2016 Apr 19;8(4):225. doi: 10.3390/nu8040225 (PMC4848693; doi:10.3390/nu8040225)
Supplement: Supplementary file 1 [file nutrients-08-00225-s001.docx]

Supplementary Materials: Nutrient Patterns and Their Food Sources in Older Persons from France and Quebec: Dietary and Lifestyle Characteristics

Benjamin Allès, Cécilia Samieri, Simon Lorrain, Marthe-Aline Jutand, Pierre-Hugues Carmichael, Bryna Shatenstein,
Pierrette Gaudreau, Hélène Payette, Danielle Laurin and Pascale Barberger-Gateau

**Table S1.** Mean daily nutrient intakes according to quartiles of component scores obtained by factor analysis—principal component analysis of nutrient intake data in 3C (*n* = 1712) from 24-h recall.

| **Mean Intake/Day** | **Healthy Pattern Score** | | | | | **Western Pattern Score** | | | | | **Traditional—South-West of France Pattern Score** | | | | |
| --- | --- | --- | --- | --- | --- | --- | --- | --- | --- | --- | --- | --- | --- | --- | --- |
|  | **Q1** | **Q2** | **Q3** | **Q4** | ***p* *** | **Q1** | **Q2** | **Q3** | **Q4** | ***p* *** | **Q1** | **Q2** | **Q3** | **Q4** | ***p* *** |
| Proteins (g) | 73.0 | 70.2 | 74.5 | 82.0 | <0.001 | 71.1 | 70.8 | 75.0 | 82.8 | <0.001 | 80.9 | 71.4 | 67.8 | 79.6 | <0.001 |
| Carbohydrates (g) | 177 | 181 | 200 | 219 | <0.001 | 236 | 199 | 180 | 162 | <0.001 | 237 | 192 | 170 | 179 | <0.001 |
| SFA (g) | 32.2 | 25.0 | 23.9 | 21.2 | <0.001 | 21.3 | 24.0 | 25.5 | 31.5 | <0.001 | 27.5 | 24.1 | 24.1 | 26.6 | <0.001 |
| MUFA (g) | 26.4 | 21.0 | 19.6 | 17.9 | <0.001 | 17.2 | 20.0 | 21.2 | 26.5 | <0.001 | 21.5 | 19.6 | 20.0 | 23.7 | <0.001 |
| PUFA-*n*3 (g) | 1.8 | 1.1 | 1.0 | 1.0 | <0.001 | 0.8 | 0.9 | 1.1 | 2.2 | <0.001 | 1.2 | 1.0 | 1.0 | 1.7 | <0.001 |
| PUFA-*n*6 (g) | 7.2 | 6.3 | 6.3 | 6.4 | 0.27 | 5.6 | 5.8 | 6.2 | 8.5 | <0.001 | 6.1 | 6.0 | 6.3 | 7.8 | <0.001 |
| Fiber (g) | 12.0 | 14.6 | 18.5 | 24.2 | <0.001 | 22.4 | 17.7 | 15.6 | 13.6 | <0.001 | 19.8 | 16.7 | 16.2 | 16.6 | <0.001 |
| Calcium (mg) | 826.2 | 801.8 | 867.4 | 946.3 | <0.001 | 736.2 | 773.0 | 872.2 | 1060.3 | <0.001 | 1090.7 | 840.9 | 730.9 | 779.1 | <0.001 |
| Phosphorus (mg) | 1063 | 1021 | 1099 | 1235 | <0.001 | 1042 | 1023 | 1099 | 1255 | <0.001 | 1263 | 1053 | 979 | 1124 | <0.001 |
| Magnesium (mg) | 218 | 229 | 259 | 312 | <0.001 | 279 | 248 | 241 | 249 | <0.001 | 291 | 243 | 231 | 253 | <0.001 |
| Potassium (mg) | 2142 | 2381 | 2803 | 3460 | <0.001 | 3090 | 2680 | 2530 | 2485 | <0.001 | 3034 | 2609 | 2461 | 2681 | <0.001 |
| Iron (mg) | 9.9 | 9.5 | 11.0 | 13.5 | <0.001 | 12.4 | 10.6 | 10.1 | 10.8 | <0.001 | 10.2 | 9.6 | 10.2 | 14.0 | <0.001 |
| Zinc (mg) | 6.5 | 6.8 | 7.5 | 8.2 | <0.001 | 8.0 | 7.1 | 6.9 | 7.2 | 0.05 | 6.5 | 7.1 | 7.2 | 8.2 | 0.001 |
| Carotene (mg) | 1489 | 2290 | 3337 | 6792 | <0.001 | 6113 | 3235 | 2634 | 1926 | <0.001 | 4110 | 3013 | 3248 | 3537 | 0.01 |
| Vitamin A (mg) | 641.3 | 577.7 | 635.9 | 868.0 | 0.43 | 995.4 | 570.1 | 440.0 | 717.5 | 0.03 | 276.0 | 266.3 | 317.8 | 1862.8 | <0.001 |
| Vitamin C (µg) | 42.7 | 67.8 | 85.2 | 134.3 | <0.001 | 120.9 | 82.0 | 69.5 | 57.7 | <0.001 | 81.9 | 75.6 | 82.5 | 90.0 | 0.007 |
| Vitamin D (µg) | 2.9 | 1.5 | 1.2 | 1.1 | <0.001 | 1.0 | 1.2 | 1.3 | 3.3 | <0.001 | 1.0 | 1.2 | 1.5 | 3.0 | <0.001 |
| Vitamin E (µg) | 5.8 | 5.9 | 6.5 | 7.7 | <0.001 | 6.4 | 6.1 | 6.2 | 7.3 | 0.06 | 6.2 | 6.3 | 6.2 | 7.2 | 0.001 |
| Vitamin B6 (µg) | 1.2 | 1.3 | 1.4 | 1.8 | <0.001 | 1.6 | 1.4 | 1.3 | 1.4 | <0.001 | 1.5 | 1.3 | 1.3 | 1.6 | <0.001 |
| Folates (mg) | 191.8 | 226.7 | 281.7 | 387.7 | <0.001 | 351.1 | 263.6 | 239.4 | 233.8 | <0.001 | 236.8 | 237.5 | 262.1 | 351.5 | <0.001 |
| Vitamin B12 (µg) | 5.5 | 5.3 | 5.1 | 6.3 | 0.38 | 5.8 | 5.0 | 4.7 | 6.7 | 0.04 | 2.7 | 2.9 | 3.6 | 13.0 | <0.001 |
| Alcohol (g) | 14.4 | 11.7 | 11.2 | 12.6 | 0.02 | 15.8 | 13.4 | 9.9 | 10.8 | <0.001 | 11.0 | 10.9 | 13.1 | 14.9 | <0.001 |
| Energy (kcal) | 1762 | 1612 | 1674 | 1751 | <0.001 | 1783 | 1671 | 1619 | 1726 | <0.001 | 1901 | 1628 | 1543 | 1726 | <0.001 |

Q: quartile. SFA: Saturated Fatty Acids. MUFA: Monounsaturated Fatty Acids. PUFA: Polyunsaturated Fatty Acids; * *p* for ANOVA test.

**Table S2.** Mean daily nutrient intakes according to quartiles of component scores obtained by factor analysis—principal component analysis of nutrient intake data in NuAge (*n* = 1596) from first 24-h recall.

| **Mean Intake/Day** | **Healthy Pattern Score** | | | | | **Western Pattern Score** | | | | | **Traditional Pattern Score** | | | | |
| --- | --- | --- | --- | --- | --- | --- | --- | --- | --- | --- | --- | --- | --- | --- | --- |
|  | **Q1** | **Q2** | **Q3** | **Q4** | ***p* *** | **Q1** | **Q2** | **Q3** | **Q4** | ***p* *** | **Q1** | **Q2** | **Q3** | **Q4** | ***p* *** |
| Proteins (g) | 74.5 | 74.7 | 77.5 | 87.3 | <0.001 | 83.8 | 73.6 | 74.1 | 82.6 | <0.001 | 67.9 | 66.9 | 77.4 | 101.8 | <0.001 |
| Carbohydrates (g) | 227.3 | 218.9 | 241.2 | 273.8 | <0.001 | 287.7 | 233.1 | 221.9 | 218.5 | <0.001 | 277.9 | 229.3 | 223.7 | 230.3 | <0.001 |
| SFA (g) | 30.7 | 23.1 | 20.6 | 19.4 | <0.001 | 19.5 | 20 | 22.8 | 31.5 | <0.001 | 24.1 | 20.2 | 22.7 | 26.8 | <0.001 |
| MUFA (g) | 31.8 | 25.5 | 24.2 | 24.5 | <0.001 | 22.4 | 22.7 | 26 | 34.9 | <0.001 | 30.7 | 23.7 | 24.2 | 27.4 | <0.001 |
| PUFA-*n*3 (g) | 1.5 | 1.4 | 1.5 | 1.8 | <0.001 | 1.2 | 1.3 | 1.6 | 2.2 | <0.001 | 2.3 | 1.5 | 1.2 | 1.2 | <0.001 |
| PUFA-*n*6 (g) | 12.4 | 10.4 | 10.2 | 9.9 | <0.001 | 8.3 | 8.9 | 10.7 | 15 | <0.001 | 15.6 | 10.1 | 8.7 | 8.5 | <0.001 |
| Fiber (g) | 13.7 | 17.6 | 21.9 | 31 | <0.001 | 26.4 | 21.4 | 19.2 | 17.2 | <0.001 | 23.6 | 20.1 | 19.6 | 21 | <0.001 |
| Calcium (mg) | 769 | 739 | 793 | 986 | <0.001 | 852 | 759 | 767 | 908 | <0.001 | 720 | 692 | 819 | 1057 | <0.001 |
| Phosphorus (mg) | 1162 | 1164 | 1302 | 1596 | <0.001 | 1398 | 1224 | 1239 | 1362 | <0.001 | 1150 | 1126 | 1262 | 1685 | <0.001 |
| Magnesium (mg) | 250 | 285 | 335 | 435 | <0.001 | 383 | 322 | 299 | 300 | <0.001 | 333 | 297 | 314 | 361 | <0.001 |
| Potassium (mg) | 2470 | 2883 | 3344 | 4270 | <0.001 | 4019 | 3206 | 2947 | 2794 | <0.001 | 3205 | 2918 | 3140 | 3704 | <0.001 |
| Iron (mg) | 12.6 | 12.3 | 13.9 | 16.5 | <0.001 | 15.5 | 13.2 | 12.9 | 13.7 | <0.001 | 14.1 | 12.5 | 12.9 | 15.7 | 0.02 |
| Zinc (mg) | 10.8 | 9.9 | 10.3 | 11.5 | 0.03 | 11.2 | 9.8 | 9.7 | 12 | <0.001 | 8.7 | 8.6 | 10.1 | 15.2 | <0.001 |
| Carotene (mg) | 2786 | 4471 | 7388 | 14027 | <0.001 | 12,390 | 7515 | 4426 | 4343 | <0.001 | 8937 | 6532 | 6414 | 6789 | <0.001 |
| Vitamin A (mg) | 1206.6 | 869.9 | 839.0 | 1151.9 | <0.001 | 1690.5 | 827.9 | 745.2 | 803.8 | <0.001 | 755.4 | 734.0 | 861.7 | 1716.3 | <0.001 |
| Vitamin C (µg) | 63.4 | 94.4 | 128 | 206.5 | <0.001 | 191.8 | 123 | 99.3 | 78.2 | <0.001 | 151 | 118 | 108 | 115.8 | <0.001 |
| Vitamin D (µg) | 5.1 | 4.5 | 5.2 | 6.9 | <0.001 | 5.3 | 4.7 | 5.2 | 6.5 | <0.001 | 3.9 | 4.1 | 4.9 | 8.8 | <0.001 |
| Vitamin E (µg) | 4.7 | 5.1 | 5.6 | 8 | <0.001 | 6.3 | 5.6 | 5.3 | 6.3 | <0.001 | 7.3 | 5.4 | 5.2 | 5.5 | <0.001 |
| Vitamin B6 (µg) | 1.4 | 1.6 | 1.8 | 2.4 | 0.09 | 2.3 | 1.7 | 1.6 | 1.5 | <0.001 | 1.7 | 1.6 | 1.7 | 2.1 | <0.001 |
| Folates (mg) | 111.2 | 79.5 | 80.5 | 72.8 | <0.001 | 72.8 | 71.5 | 87.1 | 113 | <0.001 | 112 | 80 | 76.1 | 75.6 | 0.01 |
| Vitamin B12 (µg) | 5.8 | 4.3 | 4.4 | 5 | <0.001 | 7.5 | 3.6 | 3.8 | 4.5 | <0.001 | 2.3 | 2.9 | 3.7 | 10.5 | <0.001 |
| Alcohol (g) | 6.6 | 6.7 | 5.5 | 5.5 | 0.39 | 10.3 | 6.2 | 4.3 | 3.6 | <0.001 | 6.7 | 5.6 | 5.5 | 6.5 | 0.48 |
| Energy (kcal) | 2017 | 1815 | 1852 | 1998 | <0.001 | 2055 | 1782 | 1804 | 2042 | <0.001 | 2115 | 1762 | 1796 | 2008 | <0.001 |

Q: quartile. SFA: Saturated Fatty Acids. MUFA: Monounsaturated Fatty Acids. PUFA: Polyunsaturated Fatty Acids; * *p* for ANOVA test.
